# Associations of Dietary Antioxidants and Risk of Type 2 Diabetes: Data from the 2007–2012 Korea National Health and Nutrition Examination Survey

**DOI:** 10.3390/molecules22101664

**Published:** 2017-10-05

**Authors:** Dan Yedu Quansah, Kyungho Ha, Shinyoung Jun, Seong-Ah Kim, Sangah Shin, Gyung-Ah Wie, Hyojee Joung

**Affiliations:** 1 Graduate School of Public Health, Seoul National University, Seoul 08826, Korea; quesyyedu1989@snu.ac.kr (D.Y.Q.); j015kh@snu.ac.kr (K.H.); ksacute@snu.ac.kr (S.-A.K.); 2 Department of Nutrition Science, Purdue University, West Lafayette, IN 47907, USA; tlsdud426@snu.ac.kr; 3 Department of Food and Nutrition, Chung-Ang University, Gyeonggi-do 17546, Korea; ivory8320@cau.ac.kr; 4 Department of Clinical Nutrition, Research Institute & Hospital, National Cancer Center, Goyang 10408, Korea; gawie@ncc.re.kr; 5 Institute of Health and Environment, Seoul National University, Seoul 08826, Korea

**Keywords:** antioxidants, carotene, type 2 diabetes, Korean

## Abstract

Antioxidants are suggested to decrease risk of type 2 diabetes (T2D) by preventing progressive impairment of pancreatic β-cell and endothelial function. This study was aimed to investigate the association between dietary antioxidants and risk of T2D in Korean adults based on a national representative data. A total of 24,377 adults (19–74 years) who completed one-day 24 h dietary recall and health examination were included. Dietary antioxidant intakes including α-carotene (*p* < 0.0001), lycopene (*p* = 0.0107), flavan-3-ols (*p* < 0.0001), and proanthocyanidins (*p* = 0.0075) were significantly higher in non-diabetic subjects than in diabetic subjects. After adjusting for confounding variables, the highest quartile group of α-carotene intake was associated with a 48% reduced risk of T2D in men (OR: 0.52, 95% CI: 0.34–0.80, *p* for trend = 0.0037) and a 39% reduced risk in women (OR: 0.61, 95% CI: 0.38–0.996, *p* for trend = 0.0377) compared to the lowest quartile group. Men in the highest quartile of β-carotene intake showed lower risk of T2D (OR: 0.64, 95% CI: 0.42–0.97), but no significant decreasing trend. However, the intakes of total carotenoids and other antioxidants showed no significant association with the risk of T2D. These findings suggest that a further comprehensive approach which considers overall dietary pattern is required.

## 1. Introduction

According to the World Health Organization (WHO), diabetes is a chronic disease that occurs as a result of the pancreas’s inability to produce enough insulin or ineffective use of insulin produced by the body [1]. In 2014, the prevalence of diabetes was estimated to be 8.5% worldwide [2]. It is projected that diabetes will be the seventh leading cause of deaths in 2030 [3]. Currently, more than 400 million people are living with diabetes, with the vast majority of them diagnosed with type 2 diabetes mellitus (T2D) [1]. Data from the Korean National Health Insurance Service (NHIS) indicated that, the prevalence of T2D among individuals aged 30 years and older increased from 5.6% in 2006 to 8% in 2013, with an annual increment of 0.2–0.5% [4,5]. The report further revealed that T2D increased with age in both men and women although the prevalence in men was 1.2 times higher than in women [5].

Oxidative stress, a known risk factor of T2D, is an imbalance between the production of reactive oxygen species (free radicals) and the antioxidant defense mechanism [6,7,8]. Insufficient antioxidant defense against oxidative stress has been suggested to advance the development and the progression of T2D by increasing resistance of impairing insulin action [9,10]. Sufficient intake of dietary antioxidants mainly from fruits and vegetables can provide a balanced defense mechanism against the harmful effects of reactive oxygen species [10] and may reduce the risk of diabetes [11,12,13,14] and other chronic diseases [15,16,17]. The dietary antioxidants have been reported to be associated with a decreased risk of T2D by preventing the progressive impairment of pancreatic β-cell and endothelial function [7,10,18].

Several studies have suggested that adequate intake of dietary antioxidants such as flavonoids and carotenoids may decrease the risk of T2D [14,19,20,21,22]. Other studies, however, have reported no independent associations between antioxidants and risks of T2D [23,24,25]. Limitations by different study designs, short follow-up, small sample size, and antioxidant intake variability among study participants as well as inaccurate measurement and limited adjustment of important lifestyle factors such as physical activity and smoking may partly be responsible for the inconsistencies in previous studies [14].

Few studies have investigated the association between dietary antioxidant intake and T2D in Korea. Therefore, this study sought to investigate the relationship between dietary antioxidant intake and the risk of T2D among Korean adults by using the national data from the Korea National Health and Nutrition Examination Survey (KNHANES).

## 2. Results

Table 1 indicates the general characteristics of the study subjects according to T2D status. Among a total of 24,377 Korean adults (40.1% of men and 59.9% of women), those who were classified as T2D subjects constituted 2.5% (3.4% of men and 1.9% of women). Overall, subjects with T2D were more likely to be older (*p* < 0.0001), had lower level of education (*p* < 0.0001), and had higher means of body mass index (BMI) and fasting blood glucose (*p* < 0.0001). The prevalence of T2D significantly decreased as household income increased in women (*p* < 0.0001). Among men, the percentage of subjects who did regular physical activity was significantly higher in the non-diabetic group than in the diabetic group (*p* = 0.0044). Regular alcohol consumption showed a significant difference among female diabetic and non-diabetic groups (*p* = 0.0063) but not among men.

Mean daily intakes of macronutrients and antioxidants among study participants are presented in Table 2. There was no significant difference in macronutrient intakes between diabetic and non-diabetes group in both men and women. In women, the mean dietary intakes of vitamin E and retinol were significantly higher in the non-diabetic group than in the diabetic group (*p* = 0.0090 and *p* < 0.0001, respectively). For both sexes, the mean intakes of α-carotene were significantly higher in the non-diabetic group than the diabetic group (*p* < 0.0001 for men and *p* = 0.0296 for women), whereas lycopene intake was significantly higher only in non-diabetic men than those in diabetic men (*p* = 0.0350). Among flavonoid subclasses, the intakes of flavan-3-ols and proanthocyanidins were higher in the non-diabetic subjects than diabetic subjects. Flavan-3-ols showed significant differences according to T2D status in both men (*p* = 0.0013) and women (*p* = 0.0019), whereas proanthocyanidin intake was significantly different according to the status of T2D in women only (*p* = 0.0073).

The multivariable adjusted odds ratios (ORs) and 95% confidence intervals (CIs) of T2D according to quartiles of antioxidant intakes by sex are shown in Table 3. After adjusting for potential confounding variables, subjects in the highest quartile of α-carotene density showed a 48% decreased risk of T2D in men (OR: 0.52, 95% CI: 0.34–0.80, *p* for trend = 0.0037) and a 39% decreased risk in women (OR: 0.61, 95% CI: 0.38–0.996, *p* for trend = 0.0377) compared to the lowest quartile group. With regard to β-carotene, men in the highest quartile showed a 36% decreased risk of T2D, but there was no significant decreasing trend (OR: 0.64, 95% CI: 0.42–0.97, *p* for trend = 0.0618). No significant associations were observed for vitamin C, vitamin E, and flavonoid subclasses.

We also conducted stratified analysis by several risk factors of T2D for α-carotene and β-carotene intakes (Table 4). In men, the highest quartile group of α-carotene intake was associated with a decreased risk of T2D among subjects who were younger (19–49 years) (OR: 0.42, 95% CI: 0.21–0.84), did physical activity (OR: 0.38, 95% CI: 0.18–0.79), were current smokers (OR: 0.40, 95% CI: 0.23–0.69), were regular alcohol consumers (OR: 0.50, 95% CI: 0.30–0.81) and had BMI less than 25 kg/m^2^ (OR: 0.46, 95% CI: 0.25–0.87) compared to the lowest quartile group, after adjusting for covariates. In women, high α-carotene intake was associated with low likelihood of having T2D among subjects who were not regular alcohol consumers (OR: 0.50, 95% CI: 0.26–0.93) compared to the lowest quartile group. On the other hand, the highest quartile group of β-carotene intake were inversely associated with T2D in men who were regular alcohol consumers (OR: 0.62, 95% CI: 0.39–0.99) and in women who did regular physical activity (OR: 0.46, 95% CI: 0.22–0.97) and had BMI less than 25 kg/m^2^ (OR: 0.34, 95% CI: 0.17–0.67) compared to the lowest quartile group.

## 3. Discussion

In this nationally representative data, several dietary carotenoids were associated with the risk of T2D among the Korean adult population. The mean intakes of α-carotene and lycopene were significantly higher in non-diabetic subjects than those in diabetic subjects. High α-carotene intake was inversely associated with the risk of T2D in both men and women, and high β-carotene intake, the major source of dietary carotenoids, showed a decreased risk of T2D in men, after adjusting for confounding variables. However, other dietary antioxidants intakes including vitamin C, vitamin E, and flavonoids were not associated with T2D risk.

Several prospective studies have reported inverse associations between carotenoids and T2D. However, the reported types of carotenoids with inverse associations with T2D were inconsistent by studies. Among individual carotenoids, inverse associations with T2D were found in β-carotene intake among Swedish middle-aged men [14] and Dutch middle-aged adults [20] and in β-cryptoxanthin intake among Finnish middle-aged adults [11]. Unlike the current study, previous studies reported no significant association of α-carotene intake with T2D [11,20]. Total carotenoid intake was related to a decreased risk of T2D among middle-aged adults in Finland [11], but it was not the case in Netherlands [20] or in this study. According to recent cross-sectional studies conducted in Asia, dietary total carotenoid was associated with a decreased risk of high fasting blood glucose among Korean men [26], but there was no association between carotenoid intake and the risk of fasting blood glucose among Chinese adults [27]. In addition, Lee et al. [28] reported in their cross-sectional study that carotene intake was not associated with T2D in Korean adults. However, they defined diabetes by questionnaires and measured carotene intake by food frequency questionnaires , which did not consider individual carotene intake. Thus, further prospective studies are required to examine these associations of T2D with carotenoid intake in the Korean population.

Carotenoids found in fruits and vegetables are a group of pigmented compounds with yellow, orange, and red colors that are synthesized by plants and microorganism [29]. Carotenoids are present in various forms by their molecular structures and the major carotenoids of the human body and diet are β-carotene, α-carotene, β-cryptoxanthin, lycopene, lutein, and zeaxanthin [30]. The antioxidant properties of carotenoids have been focused on β-carotene, which also has the most significant provitamin A activity [29,31,32]. However, the present study observed a stronger inverse association in α-carotene than β-carotene. Even though α-carotene and β-carotene have very similar structures with a different location of double-bond, several studies have reported that α-carotene has stronger anticancer effects than β-carotene by inhibiting proliferation of the neuroblastoma in human [33] and suppressing carcinogenesis in animal [34]. According to the third National Health and Nutrition Examination Survey Follow-up Study in the US, serum α-carotene level was inversely associated with risk of death from diabetes as well as all causes among US adult population [35]. Although mechanisms underlying different effects of α-carotene and β-carotene on chronic diseases have not been fully understood yet, our findings suggest that not only β-carotene but also α-carotene should be paid attention to in the prevention and management of T2D.

In this study, diabetic women consumed less vitamin C, vitamin E, and several subclasses of flavonoids including flavan-3-ols and proanthocyanidins than non-diabetic women. Diabetic men consumed less flavan-3-ols than non-diabetic men. However, the current study found no significant association between these dietary antioxidant intakes and the risk of T2D. Previous reports on these associations have been inconsistent. Some observational studies in the US, Finland, and China reported that dietary antioxidants such as vitamin C [11,27], vitamin E [27,36], and flavonoids [21,23] were not associated with T2D. On the other hand, the inverse association between the intakes of vitamin C in Chinese adults [36], vitamin E in Finnish adults [11], and several subclasses of flavonoids in American [22] and European adults [37] and T2D risk have been indicated. Considering that most of these previous studies used food frequency questionnaires, there may be limitation of measuring exact differences in dietary antioxidant intakes between diabetic and non-diabetic subjects. In addition, the varied range of validity and reliability of antioxidant databases by studies might partly contribute to inconsistent associations between dietary antioxidant intake and T2D.

It is well-known that the higher intakes of fruit and vegetable are associated with a reduced risk of T2D. In our additional analyses, major food items contributing to α-carotene intake in Korean adult population were carrot, laver, and pumpkin, and those contributing to β-carotene intake were radish leaves, carrot, chili pepper, perilla leaves, and laver. Recent meta-analyses reported that consumption of yellow vegetables and green leafy vegetables were associated with a decreased risk of T2D [12,38]. A decreasing trend in the risk of T2D was observed according to flavone intake among women in the present study, which shared similar vegetable sources such as chili peppers and sweet peppers [39], although the odds ratio of highest quartile group of flavone intake was not statistically significant.

Meanwhile, Chun and her colleges [40] found that fruits generally have higher antioxidant capacities than vegetables do. However, dietary antioxidants mainly in fruits were not associated with T2D in this study. This null association might be due partly to distinct dietary characteristics of the Korean population. Traditionally, the Korean diet is a rice-based high-carbohydrate diet. According to the Korea Health Statistics, more than 60% of energy was from carbohydrates. The energy contribution of carbohydrate increased by age, which is in line with an increasing prevalence of diabetes by age among Korean adults [41]. Dietary guidelines for diabetes have suggested that lower-glycemic index foods may have a positive effect on glycemic control [42]. Thus, the antioxidant effects of fruits might offset by the consumption of high-glycemic foods such as refined grains and sugars. On the other hand, antioxidant-rich fruits and vegetables contain relatively high fiber, which can influence the beneficial association with T2D [12]. Further studies are needed to investigate the interaction between dietary antioxidants and other dietary factors such as carbohydrate and fiber intakes.

A variety of factors such as age and lifestyles have been known to contribute to the development and progress of T2D. Prevalence of T2D in Koreans drastically increased by age [41]. Obesity and unhealthy lifestyles including smoking and heavy alcohol consumption have been known to influence negative effects on diabetes [43,44,45], whereas physical activity plays a favorable role in managing diabetes [46]. In accordance with our stratified analysis, the observed inverse associations between α-carotene and β-carotene and T2D were different by several potential risk factors for T2D such as age, BMI, and health-related behaviors. In men at a younger age (19–49 years), α-carotene intake was inversely associated with T2D. Among subjects who had less BMI or were physically active, high α-carotene intake of men and high β-carotene intake of women were associated with decreased risk of T2D. On the other hand, inverse associations of α-carotene and β-carotene intake were observed in men who were currently smoking and drinking alcohol regularly. Although it is difficult to explain fully why these mixed findings were found in Korean adults, these findings suggest that high α-carotene and β-carotene intake may be related to a decreased risk of T2D, especially in men with unhealthy lifestyles.

This study has several limitations. First, we cannot conclude on a causal relationship between dietary antioxidant intake and T2D due to the cross-sectional design. Second, this study included a relatively small number of diabetic subjects compared to non-diabetic subjects. When we tried to analyze using randomly selected age- and sex- matched controls, a stronger inverse association between α- and β-carotene and T2D was observed (data not presented). Further prospective studies with a large number of diabetic subjects among the Korean population are needed. Third, the use of the one-day 24 h recall data might not be sufficient enough to reflect individuals’ usual intakes due to intra-individual variability. Fourth, the incompleteness of the antioxidant database may have led to an underestimation of the antioxidant intake. However, the database covers antioxidant vitamin content for 99.6% of food consumption and flavonoid content for 78.5% of food consumption. Thus, the high coverage of database may lessen the likelihood of underestimation. Finally, the estimated antioxidant intakes might not reflect the bio-availability of each antioxidant. Despite these limitations, the present study, to the best of our knowledge, is the first to identify a relationship between dietary antioxidants and T2D based on a systemically established database in a large nationally representative sample of Korean adults.

## 4. Methods 

### 4.1. Study Design and Population

The KNHANES is a nationally representative cross-sectional surveillance system that assesses the health and nutritional status of Koreans. The survey was conducted by the Korea Centers for Disease Control and Prevention (KCDC) based on the National Health Promotion Act. The KNHANES collects information on several health and nutrition related variables including health-related behaviors, anthropometric measures and dietary intakes using a complex, multistage probability sampling design to represent the Korean population. Details of the KNHANES have been published elsewhere [47]. Among 35,017 adults who were aged 19–74 years, those who did not participate in 24 h recall (*n* = 4254), who did not have a fasting blood glucose measurement (*n* = 3656), were pregnant or lactating women (*n* = 459), whose energy intake was implausible (<500 kcal/day or >5000 kcal/day) (*n* = 657), and who were diagnosed with or receiving medication for diabetes (*n* = 1614) were excluded. Finally, 24,377 subjects aged 19–74 years were included for the final analysis.

The KNHANES was approved by the KCDC Institute Review Board and written informed consent was obtained from all subjects (IRB numbers: 2007-02CON-04-P, 2008-04EXP-01-C, 2009-01CON-03-2C, 2010-02CON-21-C, 2011-02CON-06-C and 2012-01EXP-01-2C).

### 4.2. Assessment of Dietary Antioxidant Intakes

Based on the one-day 24 h recall data from the KNHANES, individual’s dietary antioxidant intake was estimated by linking the antioxidant database for Korean foods. The antioxidant database was established for common Korean foods and was composed of 3193 food items consumed by Korean adults in the 2007–2012 KNHANES. The database included contents on 42 individual antioxidants per each food item, which were vitamin C, 4 vitamin E (α-tocopherol, β-tocopherol, γ-tocopherol, and δ-tocopherol), 6 carotenoids (α-carotene, β-carotene, β-cryptoxanthin, lycopene, lutein, and zeaxanthin), 7 flavonoid subclasses (flavonols, flavones, flavanones, flavan-3-ols, anthocyanidins, isoflavones, and proanthocyanidins) and their 31 different components. The database covers flavonoid content for 78.5% of food consumption and antioxidant vitamin content for 99.6% of food consumption. The detailed description of this database is available elsewhere [39,48]. Daily antioxidant intake was estimated as the sum of antioxidants from all foods and beverages consumed by each subject during the 24 h period.

### 4.3. Measurement of Socio-Demographic and Health-Related Variables

Socio-demographic variables, including sex, age, education level, and household income status, and lifestyle factors including BMI, physical activity, smoking habits, and alcohol consumption, were assessed by the health interview survey and were used as covariates in the present analyses. Education level was divided into four levels: “elementary school or less,” “middle school,” “high school,” and “college or more.” Household income also comprised of four categories: “low,” “middle low,” “middle high,” and “high” according to quartiles of monthly household income. Physical activity was defined as “yes” if subjects walked for at least 30 min each time five times per week. Current smoking was defined as “yes” if study participants had smoked more than 100 cigarettes in their lifetime and continues to smoke. Regular alcohol consumption was defined as “yes” for those who drank more than once a month over the past year. BMI (kg/m^2^) was calculated from height (m^2^) and weight (kg) measured in health examination survey and subjects whose BMI was more than 25 kg/m^2^ were defined as obesity according to the Asia-Pacific criteria of WHO [49].

### 4.4. Definition of T2D

T2D was defined as fasting blood glucose level ≥ 126 mg/dL based on the criteria of the Korean Diabetes Association [50]. Fasting blood glucose was estimated for subjects who fasted for at least 8 h before the examination by trained health technicians.

### 4.5. Statistical Analyses

All statistical analyses were performed with Statistical Analysis System (SAS) (version 9.4; SAS Institute, Cary, NC, USA). Strata, clusters, and survey weights were applied to all analysis to reflect complex sampling design of the national survey. We presented socio-demographic variables as a percentage (%), whereas daily macronutrient and antioxidant intakes were presented as mean and standard error (SE) according to T2D status. Differences between categorical variables were tested using chi-square test and t-test for continuous variables. We categorized subjects into four groups according to quartiles of dietary antioxidant intake by T2D status for each sex. In order to adjust for energy intake, antioxidant density—which represents daily antioxidant intake per 1000 kcal—was calculated for each subject when categorizing quartiles. Multiple logistic regression analysis was carried out to estimate ORs and 95% CIs of T2D according to quartiles of antioxidant density intake. In the multiple logistic regression model, variables including age, BMI, current smoking, education level, household income, physical activity, and regular alcohol consumption were adjusted to eliminate possible confounding effects on the association between dietary antioxidant intake and T2D. *p*-value for linear trend was estimated based on median value for each quartile group. All statistical significances were two sided and accepted at *p* < 0.05.

## 5. Conclusions

Several dietary antioxidant intakes were different by T2D status in Korean adults. High α-carotene and β-carotene intakes were associated with a reduced risk of T2D, and these associations were different according to the status of other risk factors of T2D such as sex, age, BMI, and lifestyle factors. These findings suggest that a further comprehensive approach that considers overall dietary pattern is required to establish dietary guidelines or nutrition intervention programs for the prevention and management of type 2 diabetes in the Korean population.

## Figures and Tables

**Table 1:** General characteristics of study subjects according to type 2 diabetes status ^1^.

	Total					Men					Women				
Variable	Non-Diabetes (*n* 23,774)		Diabetes ^2^ (*n* 603)		*p*-Value ^3^	Non-Diabetes (*n* 9447)		Diabetes (*n* 332)		*p*-Value	Non-Diabetes (*n* 14,327)		Diabetes (*n* 271)		*p*-Value
	*n*	%	*n*	%		*n*	%	*n*	%		*n*	%	*n*	%	
Age (years)															
19–49	13,233	68.9	200	47.1	<0.0001	5153	71.1	119	51.8	<0.0001	8080	66.7	81	38.8	<0.0001
50–64	6770	23.1	240	37.7		2661	21.8	128	35.6		4109	24.3	112	41.4	
65–74	3771	8.0	163	15.2		1633	7.1	85	12.6		2138	8.9	78	19.8	
Educational level															
≤Elementary	5194	14.4	208	26.3	<0.0001	1481	9.6	81	17.8	<0.0001	3713	19.2	127	41.0	<0.0001
Middle School	2629	9.8	92	14.5		1099	9.2	50	13.7		1530	10.4	42	16.1	
High School	8564	42.1	187	40.1		3495	43.5	112	42.8		5069	40.7	75	35.5	
≥College	7095	33.7	88	19.1		3241	37.7	68	25.8		3854	29.8	20	7.5	
Household income ^4^															
Low	3790	12.7	136	17.5	<0.0001	1371	11.3	68	15.2	0.1387	2419	14.0	68	21.7	<0.0001
Middle low	5905	26.0	179	31.4		2391	25.9	85	26.8		3514	26.2	94	39.3	
Middle high	6720	30.5	157	29.9		2741	31.5	94	32.7		3979	29.5	63	25.0	
High	6977	30.8	116	21.2		2798	31.2	75	25.3		4179	30.4	41	14.0	
Physical activity ^5^, yes	9932	43.0	230	37.6	0.0334	4172	44.9	127	35.3	0.0044	5760	41.1	103	41.5	0.9127
Current smoking ^6^, yes	6329	33.3	213	43.5	<0.0001	5428	58.6	189	63.3	0.1704	901	7.7	24	9.3	0.4342
Regular alcohol consumption ^7^, yes	12,715	60.8	327	63.8	0.2139	6933	76.6	246	80.8	0.1703	5782	44.8	81	34.6	0.0063
Obesity ^8^, yes	7103	30.1	347	59.6	<0.0001	3244	35.4	170	54.8	<0.0001	3859	24.8	177	68.0	<0.0001
	**Mean**	**SE**	**Mean**	**SE**	***p*-Value**	**Mean**	**SE**	**Mean**	**SE**	***p*-Value**	**Mean**	**SE**	**Mean**	**SE**	***p*-Value**
Body mass index (kg/m^2^)	23.5	0.0	26.1	0.2	<0.0001	24.0	0.0	25.6	0.3	<0.0001	23.0	0.0	27.0	0.3	<0.0001
Fasting blood glucose (mg/dL)	92.1	0.1	157.2	2.1	<0.0001	93.2	0.1	155.9	2.7	<0.0001	91.0	0.1	159.5	3.2	<0.0001

^1^ All analyses accounted for the complex sampling design effect and appropriate weights of the national survey. ^2^ “Diabetes” were subjects whose fasting blood glucose level was ≥ 126 mg/dL. ^3^ Continuous variables were tested using t-tests, and categorical variables were tested using chi-square tests. ^4^ Household income: Low (first quartile), middle low (second quartile), middle high (third quartile), high (fourth quartile). ^5^ Physical activity: “yes,” walking for at least 30 min each time for 5 times per week. ^6^ Current smoking: “yes,” smoked ≥ 100 cigarettes over lifetime and still smoking. ^7^ Regular alcohol drinking: “yes,” drank alcohol more than once a month over the past year. ^8^ Obesity: Body mass index ≥ 25 kg/m^2^.

**Table 2:** Mean daily intakes of macronutrient and antioxidant according to type 2 diabetes status ^1^.

	Total					Men					Women				
	Non-Diabetes (*n* 23,774)		Diabetes 2 (*n* 603)		*p*-Value	Non-Diabetes (*n* 9447)		Diabetes (*n* 332)		*p*-Value	Non-diabetes (*n* 14,327)		Diabetes (*n* 271)		*p*-Value
	Mean	SE	Mean	SE		Mean	SE	Mean	SE		Mean	SE	Mean	SE	
Macronutrient intake															
Energy (kcal/day)	2018.6	7.8	2104.0	45.4	0.0605	2346.9	11.5	2370.4	58.9	0.6929	1685.5	7.2	1633.3	50.2	0.3001
Carbohydrate (g/day)	318.6	1.2	328.6	6.6	0.1309	354.7	1.7	356.8	8.7	0.8173	281.9	1.3	278.8	8.5	0.7160
Protein (g/day)	73.4	0.4	74.5	2.1	0.5935	86.0	0.6	83.6	2.8	0.4011	60.7	0.3	58.5	2.4	0.3566
Fat (g/day)	42.6	0.3	41.3	1.9	0.4634	50.2	0.4	46.7	2.5	0.1548	34.9	0.3	31.7	2.5	0.2015
Antioxidant intake															
Vitamin C (mg/day)	109.0	1.8	116.6	8.1	0.3395	104.7	1.9	123.9	11.1	0.0849	113.3	2.3	103.6	9.1	0.2876
Vitamin E (mg α-TE/day) 3	6.6	0.0	6.4	0.3	0.4572	7.4	0.1	7.2	0.4	0.5813	5.9	0.0	5.1	0.3	0.0090
Retinol (μg/day)	114.6	2.8	103.0	13.2	0.3850	132.9	5.1	124.9	20.3	0.6993	96.1	1.9	64.3	6.5	<0.0001
Total carotenoids (μg/day)	9257.1	123.1	8867.1	452.7	0.3908	9707.2	169.4	9305.5	627.1	0.5308	8800.6	132.7	8092.6	606.7	0.2557
α-carotene (μg/day)	556.3	10.6	374.6	40.2	<0.0001	609.5	16.0	392.8	46.9	<0.0001	502.3	12.5	342.3	72.5	0.0296
β-carotene (μg/day)	3624.2	41.8	3647.2	180.9	0.8998	3944.8	54.4	3792.7	240.1	0.5370	3299.1	49.1	3390.1	312.0	0.7732
β-cryptoxanthin (μg/day)	552.0	17.9	716.3	88.6	0.0556	507.6	16.9	783.6	122.1	0.0229	597.0	24.4	597.4	91.1	0.9964
Lycopene (μg/day)	2224.5	95.1	1545.2	266.9	0.0107	2162.1	123.3	1421.4	340.1	0.0350	2287.9	99.2	1764.1	417.2	0.2136
Lutein + Zeaxanthin (μg/day)	2300.0	38.1	2583.8	206.7	0.1718	2483.2	50.4	2915.0	295.6	0.1460	2114.2	43.8	1998.6	221.0	0.6074
Total flavonoids (mg/day)	205.0	3.8	186.5	11.1	0.1069	201.8	4.8	189.9	14.2	0.4289	208.2	5.0	180.4	18.1	0.1379
Flavonols (mg/day)	29.6	0.5	32.1	2.3	0.2677	32.9	0.6	34.1	3.0	0.6933	26.2	0.6	28.6	3.7	0.5206
Flavones (mg/day)	1.4	0.0	2.4	1.1	0.3222	1.4	0.0	3.1	1.7	0.3173	1.3	0.1	1.0	0.1	0.1022
Flavanones (mg/day)	8.7	0.3	8.1	1.5	0.7059	7.9	0.4	8.1	1.8	0.9240	9.5	0.4	8.2	2.4	0.5931
Flavan-3-ols (mg/day)	24.5	1.8	12.6	1.7	<0.0001	23.8	3.0	11.9	2.1	0.0013	25.2	2.0	14.0	2.9	0.0019
Anthocyanidins (mg/day)	49.7	1.6	53.5	5.7	0.5080	46.3	1.6	52.3	7.0	0.3902	53.1	2.4	55.6	9.7	0.8045
Isoflavones (mg/day)	19.9	0.3	21.4	1.8	0.4045	23.7	0.5	24.2	2.6	0.8681	16.1	0.3	16.6	1.8	0.7856
Proanthocyanidins (mg/day)	71.3	1.7	56.3	5.5	0.0075	65.7	2.0	56.2	7.5	0.2193	76.9	2.2	56.5	7.4	0.0073

^1^ All analyses accounted for the complex sampling design effect and appropriate weights of the national survey. ^2^ “Diabetes” were subjects whose fasting blood glucose level was ≥ 126 mg/dL. ^3^ α-TE: α-tocopherol equivalents.

**Table 3:** Multivariable adjusted odds ratios (ORs) and 95% confidence intervals (CIs) of type 2 diabetes according to quartiles of antioxidant intake ^1^.

	Men					Women				
Antioxidant	Quartiles of Antioxidant Intake ^2^				*p* for Trend	Quartiles of Antioxidant Intake				*p* for Trend
	Q1	Q2	Q3	Q4		Q1	Q2	Q3	Q4	
Vitamin C										
Median (mg/1000 kcal/day)	11.6	23.2	40.3	91.6		13.1	29.2	58.7	139.5	
OR (95% CI) ^3^	1.00	1.01 (0.68–1.51)	0.71 (0.47–1.06)	1.08 (0.72–1.63)	0.6482	1.00	0.96 (0.62–1.48)	0.81 (0.51–1.28)	0.88 (0.57–1.35)	0.5488
Vitamin E										
Median (mg α-TE/1000 kcal/day) ^4^	1.5	2.3	3.2	4.9		1.5	2.5	3.5	5.5	
OR (95% CI)	1.00	0.90 (0.61–1.33)	0.78 (0.53–1.17)	0.82 (0.55–1.24)	0.3469	1.00	0.88 (0.57–1.35)	1.53 (1.01–2.32)	0.70 (0.43–1.15)	0.3960
Retinol										
Median (μg/1000 kcal/day)	1.7	15.7	40.6	94.5		1.2	16.7	45.1	107.2	
OR (95% CI)	1.00	1.25 (0.84–1.84)	0.97 (0.64–1.47)	0.96 (0.62–1.49)	0.4699	1.00	1.01 (0.68–1.50)	0.82 (0.51–1.31)	0.95 (0.60–1.51)	0.7551
Total carotenoids										
Median (μg/1000 kcal/day)	857.5	2006.1	3681.6	8317.3		953.1	2337.2	4523.7	10469.0	
OR (95% CI)	1.00	0.79 (0.52–1.20)	0.85 (0.57–1.27)	0.91 (0.60–1.37)	0.9263	1.00	1.12 (0.74–1.70)	0.67 (0.42–1.07)	0.97 (0.63–1.48)	0.6831
α-carotene										
Median (μg/1000 kcal/day)	0.0	27.7	176.4	549.2		0.0	38.6	193.5	594.2	
OR (95% CI)	1.00	0.87 (0.60–1.26)	0.84 (0.56–1.27)	0.52 (0.34–0.80)	0.0037	1.00	1.02 (0.68–1.53)	1.11 (0.72–1.72)	0.61 (0.38–0.996)	0.0377
β-carotene										
Median (μg/1000 kcal/day)	451.4	993.2	1634.7	3049.7		477.1	1045.2	1749.7	3443.6	
OR (95% CI)	1.00	0.76 (0.50–1.16)	0.91 (0.62–1.34)	0.64 (0.42–0.97)	0.0618	1.00	0.69 (0.45–1.06)	0.80 (0.51–1.24)	0.75 (0.48–1.18)	0.3914
β-cryptoxanthin										
Median (μg/1000 kcal/day)	6.3	60.9	141.0	397.3		6.0	62.8	169.6	645.8	
OR (95% CI)	1.00	0.85 (0.57–1.28)	1.03 (0.69–1.53)	1.10 (0.74–1.62)	0.4024	1.00	0.79 (0.51–1.24)	0.65 (0.41–1.03)	0.94 (0.60–1.46)	0.7855
Lycopene										
Median (μg/1000 kcal/day)	0.0	0.0	0.1	1189.8		0.0	0.0	0.2	2762.8	
OR (95% CI)	1.00	1.36 (0.70–2.63)	0.88 (0.61–1.28)	0.95 (0.65–1.38)	0.8642	1.00	0.79 (0.39–1.59)	1.04 (0.70–1.56)	0.76 (0.48–1.20)	0.2153
Lutein + Zeaxanthin										
Median (μg/1000 kcal/day)	108.3	351.3	741.7	2356.6		115.5	386.2	862.7	2572.6	
OR (95% CI)	1.00	0.99 (0.67–1.46)	0.84 (0.55–1.30)	1.11 (0.75–1.63)	0.4635	1.00	1.01 (0.64–1.60)	1.18 (0.77–1.79)	0.90 (0.60–1.35)	0.5060
Total flavonoids										
Median (mg/1000 kcal/day)	16.2	37.7	71.9	177.5		17.9	47.9	103.8	239.8	
OR (95% CI)	1.00	0.81 (0.53–1.24)	1.00 (0.68–1.46)	0.75 (0.49–1.15)	0.2759	1.00	1.30 (0.86–1.96)	0.89 (0.55–1.44)	0.97 (0.60–1.57)	0.5111
Flavonols										
Median (mg/1000 kcal/day)	2.9	7.1	11.6	24.1		2.7	6.8	11.6	24.7	
OR (95% CI)	1.00	0.69 (0.46–1.05)	0.80 (0.54–1.19)	0.79 (0.52–1.19)	0.5115	1.00	0.85 (0.54–1.32)	1.07 (0.69–1.66)	0.89 (0.56–1.43)	0.7853
Flavones										
Median (mg/1000 kcal/day)	0.0	0.3	0.6	1.3		0.0	0.3	0.6	1.4	
OR (95% CI)	1.00	0.96 (0.64–1.43)	0.76 (0.51–1.14)	0.83 (0.57–1.23)	0.3033	1.00	1.12 (0.70–1.78)	0.96 (0.63–1.46)	0.67 (0.43–1.04)	0.0256
Flavanones										
Median (mg/1000 kcal/day)	0.0	0.0	0.0	6.0		0.0	0.0	0.0	14.2	
OR (95% CI)	1.00	0.98 (0.66–1.44)	0.96 (0.66–1.41)	0.96 (0.64–1.45)	0.9207	1.00	0.91 (0.60–1.38)	0.88 (0.56–1.38)	0.91 (0.58–1.42)	0.8888
Flavan-3-ols										
Median (mg/1000 kcal/day)	0.0	0.2	1.6	12.4		0.0	0.4	3.5	18.2	
OR (95% CI)	1.00	0.68 (0.45–1.02)	0.83 (0.55–1.27)	0.67 (0.44–1.01)	0.1991	1.00	1.00 (0.66–1.50)	1.02 (0.64–1.64)	0.88 (0.54–1.42)	0.5162
Anthocyanidins										
Median (mg/1000 kcal/day)	0.0	4.1	12.1	40.4		0.1	5.4	17.0	62.2	
OR (95% CI)	1.00	0.83 (0.56–1.24)	0.97 (0.65–1.45)	1.03 (0.67–1.56)	0.5906	1.00	0.93 (0.61–1.43)	0.83 (0.53–1.28)	1.24 (0.79–1.96)	0.1960
Isoflavones										
Median (mg/1000 kcal/day)	0.4	3.7	8.8	21.9		0.2	2.7	7.7	20.8	
OR (95% CI)	1.00	0.98 (0.66–1.45)	0.77 (0.51–1.15)	0.95 (0.63–1.42)	0.7998	1.00	1.27 (0.76–2.14)	1.35 (0.82–2.24)	1.28 (0.79–2.06)	0.4672
Proanthocyanidins										
Median (mg/1000 kcal/day)	0.0	1.9	11.0	75.8		0.0	3.3	24.0	113.3	
OR (95% CI)	1.00	1.00 (0.66–1.51)	0.93 (0.63–1.37)	0.68 (0.45–1.03)	0.0422	1.00	1.49 (0.97–2.29)	1.25 (0.78–2.02)	0.92 (0.55–1.54)	0.1778

^1^ All analyses accounted for the complex sampling design effect and appropriate weights of the national survey. ^2^ Antioxidant intake were categorized into quartiles based on the antioxidant intake density (mg/1000 kcal/day). ^3^ Adjusted for age, body mass index, current smoking, education, household income, physical activity, and regular alcohol consumption. ^4^ α-TE: α-tocopherol equivalents.

**Table 4:** Multivariable adjusted odds ratios (ORs) and 95% confidence intervals (CIs) of type 2 diabetes according to quartiles of carotene intake stratified by several risk factors ^1^.

	Quartiles of α-Carotene Intake ^2^				Quartiles of β-Carotene Intake ^2^			
	Men		Women		Men		Women	
	Q1	Q4	Q1	Q4	Q1	Q4	Q1	Q4
Age ^3^								
19–49 years	1.00	0.42 (0.21–0.84)	1.00	0.40 (0.16–1.01)	1.00	0.68 (0.37–1.24)	1.00	0.56 (0.24–1.31)
50–74 years	1.00	0.66 (0.39–1.09)	1.00	0.78 (0.45–1.35)	1.00	0.62 (0.36–1.06)	1.00	0.85 (0.51–1.44)
Physical activity ^4,5^								
Yes	1.00	0.38 (0.18–0.79)	1.00	0.70 (0.32–1.55)	1.00	0.62 (0.34–1.13)	1.00	0.46 (0.22–0.97)
No	1.00	0.64 (0.38–1.09)	1.00	0.57 (0.29–1.10)	1.00	0.64 (0.38–1.09)	1.00	1.07 (0.60–1.92)
Current smoking ^6,7^								
Yes	1.00	0.40 (0.23–0.69)	NE ^8^	NE	1.00	0.82 (0.49–1.38)	NE	NE
No	1.00	0.71 (0.35–1.45)	NE	NE	1.00	0.60 (0.32–1.11)	NE	NE
Regular alcohol consumption ^9,10^								
Yes	1.00	0.50 (0.30–0.81)	1.00	0.63 (0.27–1.48)	1.00	0.62 (0.39–0.99)	1.00	0.67 (0.29–1.51)
No	1.00	0.74 (0.31–1.77)	1.00	0.50 (0.26–0.93)	1.00	0.62 (0.25–1.52)	1.00	0.83 (0.48–1.42)
Body mass index ^11^								
<25 kg/m^2^	1.00	0.46 (0.25–0.87)	1.00	0.48 (0.22–1.05)	1.00	0.69 (0.37–1.27)	1.00	0.34 (0.17–0.67)
≥25 kg/m^2^	1.00	0.64 (0.37–1.11)	1.00	0.62 (0.34–1.14)	1.00	0.62 (0.36–1.07)	1.00	1.02 (0.58–1.81)

^1^ All analyses accounted for the complex sampling design effect and appropriate weights of the national survey. ^2^ Antioxidant intake were categorized into quartiles based on the antioxidant intake density (mg/1000 kcal/day). ^3^ Adjusted for body mass index, current smoking, education, household income, physical activity, and regular alcohol consumption. ^4^ Physical activity: “yes,” walking for at least 30 min each time for 5 times per week. ^5^ Adjusted for age, body mass index, current smoking, education, household income, and regular alcohol consumption. ^6^ Current smoking: “yes,” smoked ≥ 100 cigarettes over lifetime and still smoking. ^7^ Adjusted for age, body mass index, education, household income, physical activity, and regular alcohol consumption. ^8^ NE: not estimable due to insufficient sample size of current smoking women. ^9^ Regular alcohol drinking: “yes,” drank alcohol more than once a month over the past year. ^10^ Adjusted for age, body mass index, current smoking, education, household income, and physical activity. ^11^ Adjusted for age, current smoking, education, household income, physical activity, and regular alcohol consumption n.
